# Silence is Golden: Effect of Encouragement in Motivating the Weak Link in an Online Exercise Video Game

**DOI:** 10.2196/jmir.2551

**Published:** 2013-06-04

**Authors:** Brandon C Irwin, Deborah L Feltz, Norbert L Kerr

**Affiliations:** ^1^Department of KinesiologyKansas State UniversityManhattan, KSUnited States; ^2^Michigan State UniversityDepartment of KinesiologyEast Lansing, MIUnited States; ^3^Michigan State UniversityDepartment of PsychologyEast Lansing, MIUnited States; ^4^University of KentCanterburyUnited Kingdom

**Keywords:** active video games, physical activity, exercise, Köhler effect, partner exercise, social influence, group dynamics, group exercise, virtual partner, intensity, duration, online video games

## Abstract

**Background:**

Despite the physical and mental health benefits, few adults meet US Department of Health and Human Services physical activity guidelines for exercise frequency, intensity, and duration. One strategy that may increase physical activity duration is exercising with an Internet partner (ie, someone who is virtually present, as in video chat). Internet partners help people overcome many barriers associated with face-to-face exercise groups (eg, time, coordinating schedules, social physique anxiety). Past research examining individual performance in groups suggests that an increase in effort occurs when performing a task conjunctively, ie, when a participant is (1) less capable than fellow group members, and (2) participants efforts are particularly indispensable for group success (ie, where the group’s potential productivity is equal to the productivity of its least capable member). This boost in effort is more commonly known as the Köhler effect, named after the German psychologist who first observed the effect. While encouragement between group members is common practice in face-to-face group exercise, the effect of encouragement between partners exercising conjunctively across the Internet is unknown.

**Objective:**

To examine the impact of exercising alone, compared to exercising conjunctively with an Internet partner, both with and without encouragement, on exercise persistence (primary outcomes) and secondary psychosocial outcomes (self-efficacy, enjoyment, exercise intention).

**Methods:**

Participants were recruited online and face-to-face from the campus of Michigan State University. With the assistance of the experimenter, participants (n=115) played an exercise video game in a laboratory, performing a series of five abdominal plank exercises where they were asked to hold the plank for as long as possible (Time 1). They were then randomized to a condition (Individual, Partner-without-encouragement, or Partner-with-encouragement), where they performed the exercises again (Time 2). The impact of condition on the primary outcome measures and secondary outcome measures were evaluated using a 2 (Gender) x 3 (Condition) ANOVA on change scores (Time 2-Time 1).

**Results:**

Those who exercised in online teams (n=80) exercised significantly longer (time=78.8s, *P*<.001) than those who worked individually (n=35). However, exercise duration was shorter when one’s more capable partner gave verbal encouragement (n=55) than when s/he did not (n=25) (a mean difference of 31.14s). These increases in effort were not accompanied by altered task self-efficacy, enjoyment of the task, or intention to exercise in the future.

**Conclusions:**

Exercising conjunctively with an Internet partner can boost one’s duration of exercise. However, encouragement from the stronger to the weaker member can mitigate these gains, especially if one perceives such comments being directed at someone other than themselves. To boost exercise duration, Internet-based physical activity interventions involving group interaction should make relative abilities of participants known and communication clear.

## Introduction

Despite the links between regular physical activity and positive health benefits [1-7], less than 5% of US adults achieve recommended levels of intensity and duration [1-8]. Recent strategies to promote physical activity have harnessed the Internet, a medium through which the delivery of interventions can be highly cost-effective, with the potential to reach and impact a wide audience [9]. However, Internet-delivered interventions typically produce only small effect sizes [10] and, thus, may benefit from supplemental strategies to increase the intensity and duration of physical activity.

Recent research has highlighted the influential role of social factors in physical activity behavior, including exercising with a partner [9,11]. When exercising under the right conditions, exercise partners have been shown to increase the intensity and duration of exercise by up to 208% [12,13]. Importantly, exercising with a partner affords the opportunity to encourage one another, which may further increase the duration of exercise. However, few, if any, Internet-delivered interventions create opportunities for exercising in real-time with other exercise participants.

The purpose of the current study was to test the efficacy of an Internet partner in increasing exercise duration. Specifically, we tested whether being the “weak link” on a team with an Internet partner could motivate one to exercise longer than when exercising alone. Further, we tested whether encouragement from the partner could motivate one to exercise longer still.

Basic behavioral research in social psychology suggests that the presence of a superior partner may motivate one to exercise longer than they would when exercising alone and further still if one is on a team with that partner and is the team’s “weak link” [14]. This motivation-boosting phenomenon among the team’s weak link has been coined the Köhler effect, named after the German industrial psychologist who first observed the effect in his seminal work [15,16]. In light of this evidence, we chose to make participants the weak link between them and the Internet partner and hypothesized that this would lead to exercising longer than when exercising alone. Although no previous studies on the Köhler effect have examined the influence of encouragement on the duration of exercise in the weak link, we reasonably hypothesized that encouragement would further boost exercise duration.

## Method

### Design

The study used a randomized trial in a 3 (condition: individual control, partner with- encouragement, partner-without-encouragement) x 2 (performance block: Block 1 & Block 2) factorial design. Eligible participants were students who had no physical injuries that would prevent or obstruct their performance during an isometric plank exercise.

### Setting

The study was conducted in a laboratory on the campus of Michigan State University in the Departments of Kinesiology and Psychology and was approved by the Institutional Review Board. Most of the data for two of the conditions (ie, 26 participants in the individual condition and 47 in the partner-without-encouragement condition) were collected as part of a study [17] completed about the time we decided to examine the effect of verbal encouragement (August 2009 to May 2010). In a new wave of data collection (August-December, 2010), we not only collected data for the partner-with-encouragement condition, but also some additional data in the two other conditions. By contrasting the latter with those collected for the aforementioned study [17], we could probe for possible history or cohort effects. We did not expect any systematic differences between the two waves of data collection because the lab settings, participant populations, and procedures for both data collection periods were identical, except for the introduction of the manipulation of verbal encouragement.

### Exercise Task

The task for this study was an abdominal plank exercise. These are body-weight resistance exercises where participants suspend their own body weight using their abdominal muscles. These exercises are also isometric in nature, require very little coordination, and are highly effort-based. Each exercise targeted the abdominal muscles, but there were slight differences between each (eg, holding a push-up position on one’s forearms vs on each side; a detailed description of all exercises is provided elsewhere [12]).

The exercises were performed as part of an exercise video game designed for the PlayStation 2 (PS2) gaming system as used in a previous study [12]. The software used was EyeToy: Kinetic, a game that offers a variety of fitness activities (eg, yoga, strengthening exercises, combat exercises). This particular software operates in conjunction with an additional accessory called the Eye Toy, designed specifically for the PS2 system. The Eye Toy is a small camera that connects to the PS2 system via a USB cable and allows images of the user to be displayed on the TV monitor and interact with objects in a virtual environment supported by the software.

### Procedure

A detailed description of experimental procedures is provided elsewhere [12]. In the current study, we simply describe the basic procedure and note how the encouragement manipulation was achieved.

After arriving at the laboratory, participant consent was obtained and all were ensured of the confidentiality and voluntary nature of their participation. Participants then watched a brief instructional video from the PS2-Eye Toy: Kinetic software in which a virtual trainer demonstrated the five exercises. A baseline measure of self-efficacy was then taken using an online questionnaire completed in the laboratory. Participants were asked throughout the session if they understood all instructions and, if not, the experimenter addressed his/her questions. All participants then performed the first block of exercises, holding each of the five exercises for as long as they could and with 30s rest periods between each exercise. Immediately after each exercise, the participant announced his/her perceived exertion. All participants were given veridical feedback on their performance (ie, the average of the number of seconds they held each exercise).

The condition manipulation was introduced at this point. Participants in the individual control conditions simply rested for 10 minutes. Participants in the partner conditions were told that another participant was being run simultaneously at another lab on campus and that the 2 participants would be able to see one another over an Internet video connection during future trials. The participants then met briefly with that other same-sex participant in a controlled Skype-like interaction (we will refer to that other participant hereafter as “the partner”). In reality, the partner was an experimental confederate whose side of the interaction was prerecorded. After the interaction, participants were also given bogus feedback on how well the partner had done on the first trial. That feedback score was manipulated to be 1.4 times the participant’s own actual performance. This discrepancy was chosen based on previous research that suggests that this moderate discrepancy leads to the greatest increases in exercise duration (ie, the greatest Köhler effect) [18,19].

Participants were told that they and their partners would be a 2-person exercise team. In the encouragement condition, both teammates were told that they would have the opportunity to communicate with each other during the exercises but that, due to technical problems, this capability would be provided only for the partner. Thus, ostensibly both could speak, but only the partner would be heard by the subject. No mention was made of any audio link between partners in the no-encouragement condition. For both partner conditions, it was further explained that they were working towards a team score, where the team score would be the persistence score of the first teammate to quit an exercise (ie, as soon as either partner quit, the exercise was over). This task structure is more commonly known as a conjunctive task—when the group’s performance is defined by the performance of the least-capable member (ie, the “weak link”). Following these instructions, all participants responded again to the self-efficacy measure.

Block 2 then commenced. In the individual control condition, the participants could only see him/herself on the screen, as during Block 1. In the partner conditions, the participant could see the partner’s image (which was actually prerecorded) before and during the exercise; the participant believed that the partner could likewise see his/her (the participant’s) image. The images available to the participant suggested that s/he was always the first to quit each exercise. The video link was allegedly frozen as soon as either teammate quit an exercise and until just before the start of the next exercise; hence, the participant knew only that his/her partner had been able to persist longer, but not just how much longer. In the encouragement condition, a prerecorded series of phrases of encouragement was played through a set of computer speakers controlled by the experimenter. The phrases were audible approximately every 15s (±3s) and followed the following fixed progression: “you can do it”, “you got this”, “keep it going”, “you’re doing good”, “stay strong here”, “give it your best”. After Block 2 was over, the participant completed a series of questionnaires (self-efficacy, intention to exercise, enjoyment of physical activity, and manipulation checks). S/he was then debriefed, thanked, and excused.

### Measures

#### Duration of Exercise

Duration of exercise was measured via the total number of seconds that the exercise was held. Block scores were calculated by taking the summed total of the five exercises within each trial.

#### Self-Efficacy

Task self-efficacy (SE) was measured with a scale developed specifically for this program of research using an online questionnaire. The measures contained five items, each corresponding to one of the five exercises within each trial. All items were preceded by the stem “What is the number of seconds that you are completely confident you can hold” followed by “The first exercise”, “the second exercise”, and so on for each of the five exercises. Respondents wrote in the number of seconds in a blank box following each item. The questionnaire was administered at three time points: once before Block 1 (after the participant had watched a brief instructional video demonstrating the exercises), a second time after Block 1 but before performing the five exercises at Block 2, and a third time after Block 2. A total SE score for each trial was calculated by taking the sum of the five items within each trial.

#### Ratings of Perceived Exertion

Ratings of perceived exertion (RPE) was measured using the 6-20 version of the Borg RPE scale [20]. The scale ranges from 6-20 where 6 means “no exertion at all” and 20 means “maximal exertion”. Participants were asked to verbally rate their exertion at the end of each exercise, with particular reference to their perceived exertion at the moment right before the end of the exercise.

#### Task Enjoyment

Task enjoyment was measured using a short 8-item version of the Physical Activity Enjoyment Scale (PAES) using an online questionnaire [21]. Each item was rated on a 7-point bipolar scale beginning with the stem “Please rate how you feel at the moment about the physical activity you have been doing according to the following scales” (eg, 1=“I loved it”; 7=“I hated it”). Previous studies have shown high correlations with the longer, 18-item scale (*r*=.94) [22] and strong reliability (Cronbach alpha=.91) [23].

#### Intention to Exercise

Adapted from another measure [24], intention was assessed with a single item, “I intend to exercise tomorrow for at least 30 minutes” on a scale of -3 (“Not at all true for me”) to +3 (“Completely true for me”).

#### Postexperimental Questionnaire

Besides some questions checking participants’ understanding of the instructions and procedures, there were questions probing their perceived task ability, a rating of task difficulty, and a rating of effort expended on the task, each made on 8-point scales. Participants in the partner conditions were also asked to rate their partner’s relative ability on a 9-point scale (where 1=“I am much more capable” and 9=“my partner is much more capable”).

### Plan of Analyses and Participants

Following the methods of analyses done in earlier studies, exercise duration was taken as the sum of the time each participant held the five exercises within each block, producing a Block 1 and Block 2 score. There are, of course, individual differences in fitness, intrinsic task interest, and strength that we wished to control for. This can be done in different ways. In many prior studies [14,25], participants’ Block 1 performance has been used as a baseline and difference scores (ie, Block 2-Block 1) were the primary dependent variable, one that expressed each participant’s Block 2 score relative to his/her Block 1 score (the latter baseline score reflected individual differences in fitness and strength). An alternative (less vulnerable to certain problems that can arise from the use of difference scores, eg, [26]) is to use Block 1 scores as a covariate in the analysis of Block 2 scores. Here, we present the results using the former difference-score method because the mean values presented for such an analysis are more directly understood and interpreted than means adjusted for a covariate. But the reader should note that an identical pattern of results is obtained with either method.

The analyses of the exercise duration data was to proceed in two stages. The first was to check whether there were any history or cohort effects attributable to the interval between the two data collection waves. It employed a 2 (Condition: Individual vs partner-without-encouragement) x 2 (Sex) x 2 (Data collection wave: Early vs Late) ANOVA on exercise duration difference scores (ie, Trial 2 duration-Trial 1). Although overall sex differences in the magnitude of the Köhler effect are rare, some interesting sex effects have been reported, eg, [25]; hence, participant sex was included as an experimental factor. This stage 1 analysis would permit both confirming with the larger dataset that the Köhler effect originally reported in Kerr et al [17] and checking whether its magnitude differed between the participants drawn from Kerr et al [17] and those newly recruited in the same experimental conditions for the present study. The second stage was designed to examine the primary question addressed by this paper—would verbal encouragement to the weaker partner alter the Köhler effect? If there were no data-wave moderation effects, as anticipated, we planned to drop the data-wave factor and add the no-encouragement condition in a 3 (Condition: Individuals, Partner-without-encouragement, Partner-with-encouragement) x 2 (Sex) ANOVA on exercise duration difference scores.

Although our primary focus was on exercise duration, we also examined several other variables, primarily to determine if encouragement altered any of the effects previously observed [12,17] when no encouragement was provided. For those variables collected at the end of the study (ie, task effort, intent to exercise, own task ability), the analyses would employ 3 (Condition) x 2 (Sex) analyses of variance; since the individual participants had no partner, this became a 2 (Condition: Encouragement vs No-encouragement) x 2 (Sex) ANOVA for ratings of own ability relative to one’s partner. For those variables (ie, perceived effort, self-efficacy) collected during the exercise trials, a within-Ss Block factor was added to the ANOVA. Finally, a covariate (ie, the pre-exercise estimate of task self-efficacy) was included in the analysis of self-efficacy.

## Results

**Figure 1:**
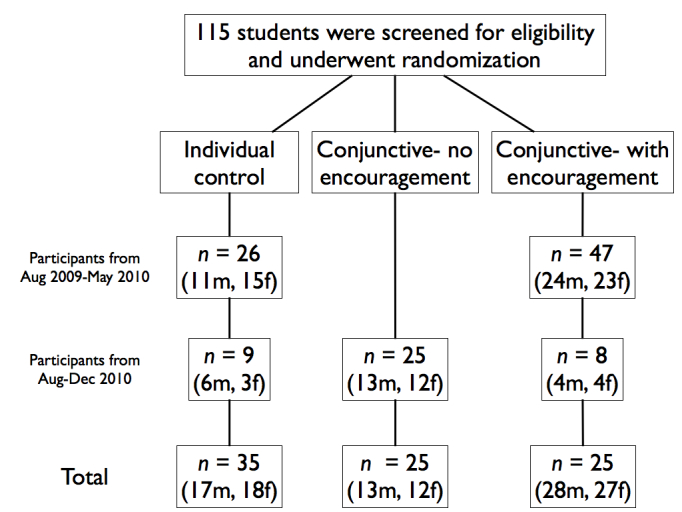
Participant flow.

Students were recruited from introductory psychology (online) and kinesiology courses (online and face-to-face) at a large Midwestern university and were given course credit for their participation. Students were recruited based on their interest in getting a good workout and were told that they would be playing an exercise video game and performing abdominal plank exercises for as long as they felt comfortable. The final total sample consisted of 115 participants (58 male, 57 female) college students (mean age 20.31, SD 3.26; see Figure 1). No participants dropped out of the study before completing their session.

As noted earlier, the participant pool and methods of recruitment were identical for the two waves of data collection, and participants were randomly assigned to conditions (single blind) within each wave using a randomization function on Microsoft Excel generated by one of the primary investigators. Hence, we expected rough equivalence in participant characteristics across waves and conditions. This could be checked for the two characteristics collected in the study—age and year in school. As expected, when the five combinations of wave and condition were compared in analyses of variance, there was no hint of between wave/condition differences (all *P*s>.15). Overall, the average participant was a sophomore/junior (mean of 2.45 where 1=1^st^ year, 2=2^nd^ year, etc) aged 20.3 years (SD 3.3).

### Exercise Duration

The initial stage 1 analyses looked for possible history or cohort effects attributable to the interval between the two data collection waves. A boost in exercise duration (ie, the Köhler effect) was evident (Condition main effect *F*_1,79_=39.53, *P*<.001, partial eta squared=.33); whereas due to fatigue, Individual condition participants persisted 39.4s less at Block 2 than at Block 1, and participants in the Partner-without-encouragement condition persisted 61.0s longer, a Köhler effect of 100.4s. More importantly, there were no significant effects involving the wave factor. Thus, the magnitude of the Köhler effect in the absence of partner encouragement was comparable within each wave of data collection. Hence, the data for these two waves were combined in all subsequent analyses.

**Figure 2:**
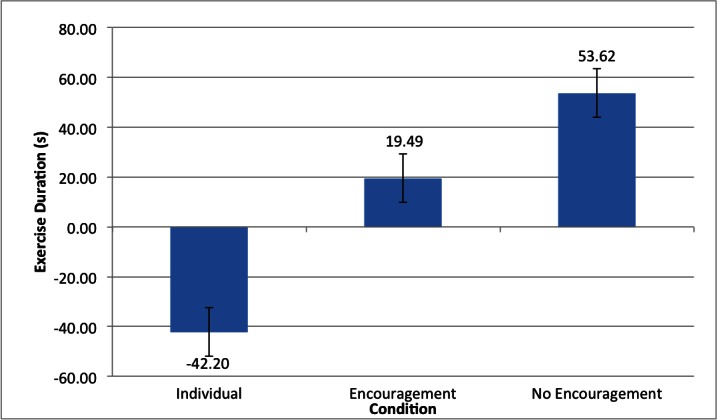
Condition Block 2 - Block 1 exercise duration means.

In the primary stage two analyses, the data for the Partner-with-encouragement condition was included to see whether and how adding verbal encouragement might affect the duration-boosting effect. The 3 (Condition: Individuals, Partner-without-encouragement, Partner-with-encouragement) x 2 (Sex) ANOVA on difference scores resulted in only one significant effect: the Condition main effect, *F*_2,105_=28.79, *P*<.001, partial eta squared=.35. Again (see Figure 2), the Individuals showed a decline in exercise duration across blocks (estimated marginal mean of -42.2s, SD 54.2), whereas partners exercised longer in the second block, whether there was (19.49s, SD 59.30s) or was not encouragement (53.63s, SD 58.44). Post hoc Dunette tests indicated that the difference between Individuals and Partners was significant (*P*<.001) in both partner conditions. A Newman-Keuls post hoc test indicated that this effect was significantly smaller (34.1s) in the Partner-with-encouragement condition.

### Ancillary Analyses

#### Exercise Self-Efficacy

PostBlock efficacy judgments (sum of number of seconds participants estimated they could persist at all 5 exercises) were examined in a 2 (Block: postBlock 1, postBlock 2) x 3 (condition) x 2 (sex) ANCOVA, which used the preBlock 1 SE score as a covariate (ie, the participant’s estimated persistence at the five exercises collected prior to the first block of exercises). The latter should reflect and control for chronic individual differences in perceived self-efficacy at such exercises. As in previous studies, there was a Block main effect, *F*_1,98_=13.74, *P*<.001, partial eta squared=.12. Participants were less sanguine about their prospects for persisting after Block 2 (adjusted mean=149.8s; SD 83.53) than after Block 1 (adjusted mean=188.4s; SD 104.1). Although condition affected actual persistence, it showed no effects on participant’s expectations of what they could do in the future.

#### Subjective Effort

Two variables were relevant here—ratings of perceived exertion (RPE), averaged across exercises within blocks (for Block 1 alpha=.93, for Block 2 alpha=.96), and the postexperimental rating of effort expended at the task. A 2 (sex) x 3 (work condition) x 2 (block) analysis of the RPE data found that participants reported greater exertion at Block 2 (14.75; SD 1.97) than Block 1 (13.88, SD 1.89; *F*_1,104_=74.34, *P*<.001, partial eta squared=.42), as one might expect given the fatiguing nature of the task. This effect was also stronger in the partner conditions (block difference mean of 1.33 in the encouragement condition and .93 in the no-encouragement condition; these did not differ significantly, ie, *P*=.11) than in the individual controls (.36; Block x Condition *F*_2,104_=6.76, *P*<.01, partial eta squared=.12), in line with the objective persistence results. A 3 (condition) x 2 (sex) ANOVA on ratings of how much effort had been expended, collected at the end of the experiment, produced no significant results.

#### Task Evaluation

An overall task enjoyment measure was computed based on the 8 items of the PAES scale (alpha=.86). The grand mean (4.68, SD .97, on the 8-point scale) was not significantly different from the scale midpoint (4.5), suggesting that participants were, at worst, indifferent toward the exercise task. There were no significant effects in a 3 (condition) x 2 (sex) ANOVA of this measure—working harder at the task with a partner did not appear to undercut participants’ enjoyment of it. Participants’ postexperimental rating of the difficulty of the task suggested that they viewed it as moderately difficult (grand mean 4.90, SD 1.65, significantly above the scale midpoint, *P*<.05). There was also a significant condition main effect on this measure, *F*_2,100_=3.63, *P*<.05, partial eta squared=.07). Post hoc Scheffé contrasts indicated that the task was rated as significantly (*P*<.05) more difficult in the no-encouragement condition (5.27)—where actual effort had been maximal—than in the individual condition (4.34), with the encouragement condition falling in between (5.10) and differing from neither of the remaining conditions.

#### Intention to Exercise

Overall, at the end of the experimental session, participants expressed a positive intent to exercise for at least 30 minutes the following day. The grand mean was 1.61 (SD 1.70) on the 7-point scale anchored by -3 (“Not at all true for me”) to +3 (“Completely true for me”); this was significantly (*P*<.05) above the midpoint of the scale. However, there were no significant effects in a 3 (condition) x 2 (sex) ANOVA of these intentions.

#### Perceptions of Task Ability

In the final questionnaire, participants were asked to rate their own ability (on an 8-point scale) and in the partner conditions, to rate their partner’s relative ability (on a 9-point scale where 0=“Not applicable. I didn’t have a partner”, 1=“I was much more capable” and 9=“My partner was much more capable”). A 3 (condition) x 2 (sex) ANOVA of own ability ratings produced a significant condition effect, *F*_2,102_=6.36, *P*<.01, partial eta squared=.11. After receiving consistent feedback indicating that they were inferior to their partner, participants in the partner conditions felt less capable (encouragement=5.26, no-encouragement=5.16) than the individuals (6.52) who had no partner. A Newman-Keuls post hoc test indicated that the two partner conditions did not differ significantly, so receiving encouragement from a partner did not alter participants’ sense of their own task ability.

However, the encouragement manipulation did affect participants’ perceptions of their partner’s ability. A 2 (encouragement vs no-encouragement) x 2 (sex) ANOVA of partner relative ability ratings revealed a strong condition effect, *F*_1,71_=10.18, *P*<.01, partial eta squared=.13. Participants in the no-encouragement condition reported that their partner was significantly more capable (7.15) than those in the encouragement condition (5.60); the latter mean did not differ significantly from the scale midpoint of 5.0, a rating that signified equal capability between teammates. This condition effect was also qualified by participant sex, sex x condition *F*_1,71_=6.98, *P*<.02, partial eta squared=.09; it was considerably stronger for males (condition difference of 2.87 for males vs .27 for females). Overall, females rated their partner’s ability higher (7.14) than males did (5.60), with sex main effect *F*_1,71_=9.72, *P*<.01, partial eta squared=.12.

## Discussion

The purpose of this study was to test the efficacy of a superior Internet partner (with and without encouragement) as a strategy for increasing the duration of exercise. Consistent with previous studies [12,13], after controlling for individual differences in strength, participants who simply exercised with a partner as the weak link persisted longer (on average, 78.8s) than those who exercised by themselves, a gain of 33.6%. This is a considerable gain for those performing a strenuous, isometric exercise where the goal is to increase strength and for those striving to meet national physical activity recommendations [27]. Furthermore, and although one might reasonably expect encouragement to boost effort at the task, we found that such encouragement mitigated*,* but did not eliminate, the effort-boosting effect of being the weak link. These findings suggest that being the weak link with a superior Internet partner may be a useful strategy for increasing exercise duration, but that at least some forms of encouragement from a superior partner may only be minimally motivating compared to exercising alone and, compared to exercising with a moderately-superior partner, may actually be de-motivating.

The fact that encouragement attenuated the duration-boosting effect of being the weak link could be explained by a few competing explanations. First, at the outset of the study, we reasoned that receiving positive encouragement could bolster one’s self-efficacy [28] and/or perceived ability and that this increase could have one of two effects on exercise duration. First, a boost in self-efficacy could lead to increases in effort, as has been shown in a number of performance-based studies [29,30]. However, we found no boost in self-efficacy in either partner condition. Conversely, we reasoned that efficacy-boosting feedback could also undermine effort, either by being so inconsistent with actual relative performance that one gives up or by leading to an overly confident belief in one’s capabilities and thereby undermining the desire to compare favorably with one’s partner [31]. Indeed, we observed significant differences in effort between the two partner conditions. However, no such increases in self-efficacy or perceived ability were found between the two partner conditions. Thus, changes in self-efficacy/perceived ability did not likely account for the attenuation of the duration-boosting effect in the encouragement condition. That encouragement did not have an effect on one’s self-efficacy does not come as a surprise, as feedback regarding one’s own performance (ie, mastery experiences) is typically more influential than verbal encouragement in affecting one’s judgments of his/her abilities [28]. Thus, it is likely that feedback indicating the partner’s superiority on Block 1 and the constant and veridical performance feedback indicating that subjects were being outperformed by their partner on Block 2 overrode or diluted any potential efficacy-boosting effect of positive encouragement offered by the partner.

Second, it was also possible that receiving encouragement from a superior other might be interpreted as condescending or patronizing [32], which could result in negative judgments of one’s partner or aversion to the task. Unfortunately, in the current study, we did not take any explicit measures of the subjects’ interpretations of the partner’s statements. However, subjects who received encouragement enjoyed the task equally as much as those who did not receive encouragement. Thus, the differences in effort between the two conditions was likely not due to the subjects’ interpretations of the encouragement as unfavorable, but clearly future studies should measure judgments of one’s partner to further and more explicitly explore this possibility.

One last possibility was that encouragement from a superior partner would be interpreted not as teammate support, but rather a method of self*-*encouragement. This could be expected especially if the intended target of the partner’s message was somewhat ambiguous, as could be the case in the current study (eg, “You can do it”; where “you” could be taken to be directed at the partner or one’s self). Interpreting the message as self-encouragement might suggest to the participants that the supposedly superior partner was in fact struggling with the task, thereby creating doubt in the degree of the partner’s superiority. When one believes that his/her partner’s ability is equal to or only slightly greater than one’s own, there may be little to be learned or gained by trying to match the partner’s performance [18]. In the present study, despite all the performance feedback suggesting that their partner was superior in ability, by the end of the experiment, those in the encouragement condition perceived themselves as comparable to their partner in ability. This strongly suggests that participants in this condition took their partner’s verbal comments (eg, “you can do it”) not as encouragement directed from a more to a less capable partner, but rather as signs that the partner was him/herself struggling with the task and was engaging in self-encouragement.

There are a number of implications of this study for physical activity promotion. First, while many studies have shown the effectiveness of group-based approaches to physical activity promotion [33], few have systematically looked at the basic underlying mechanisms behind this strategy. In this study, we do so and identify with whom (a moderately superior partner) and under what conditions (when one is the “weak link”; exercising with an Internet partner; no encouragement) exercising in groups can boost one’s effort and ultimately the duration of exercise. Second, as more physical activity promotion strategies are moving to digital modes of delivery, this study contributes to a body of basic research that provides fertile ground on which to base algorithms for electronically mediated, group-based physical activity interventions (eg, customized computer-generated partners, match-making applications). The current study suggests that when communication is part of such a partner- or group-based intervention, designers should facilitate clear and unambiguous communication between partners (eg, constrain communication to have the desired effort-boosting effect, perhaps by forcing partners to select from a fixed set of text messages).

Of course, this study has its limitations. The study was conducted in a highly controlled laboratory setting, and it may be premature to suggest that these findings generalize to free-living conditions and other populations who are more physically inactive. Second, subjects were recruited through both face-to-face and web-based systems, and because we did not code participants for how they were recruited, we were unable to control for any individual differences between recruitment strategy (eg, being recruited via Internet may have selected for people who are more motivated to work out alone). Third, although we found that being the weak link can motivate participants to exercise longer, it is not clear if the same strategy could have the same positive impact on other dimensions of physical activity behavior (eg, frequency and intensity of exercise). Last, we tested participants in only one session of exercise, and repeatedly being the “weak link” over several sessions may actually be de-motivating [34]. We should note, however, that some of the potential limitations have been addressed in other studies. For instance, researchers have recently observed the effort-boosting effect of a superior partner in other physical activity tasks and conditions, such as duration of aerobic exercise [13] and performance in swimming relay competitions, respectively [35]. Further, there is evidence to suggest that being the weak link over several exercise sessions can actually strengthen the effect, leading to increasingly longer bouts of exercise with each successive bout [13].

### Conclusion

The current study corroborates a growing body of research, which shows that exercising with a moderately superior Internet partner as the weak link can boost effort and lead to longer bouts of exercise [12,13], whether or not partners communicate with one another. However, such effort-boosting effects can be mitigated when superior partners try to encourage weaker group members, especially if this encouragement undermines the weaker member’s perception of the superior ability of his/her partner. Future research should examine the effects of a wider range of messages, exercise tasks, and conditions to help inform the design of group-based, electronically mediated physical activity interventions.

## Abbreviations

PAES: Physical Activity Enjoyment Scale
RPE: ratings of perceived exertion
SE: self-efficacy

## Multimedia Appendix 1

CONSORT-EHEALTH checklist V1.6.2 [36].
