# Supplementary material for: Silence is Golden: Effect of Encouragement in Motivating the Weak Link in an Online Exercise Video Game
Source: J Med Internet Res. 2013 Jun 4;15(6):e104. doi: 10.2196/jmir.2551 (PMC3713945; doi:10.2196/jmir.2551)
Supplement: Supplementary file 1 [file jmir_v15i6e104_app1.pdf]

CONSORT-EHEALTH Checklist V1.6.2 Report

(based on CONSORT-EHEALTH V1.6), available at [http://tinyurl.com/consort-ehealth-v1-6].

2551

Date completed

4/1/2013 11:47:18

by

Brandon Irwin

You can do it!: The efficacy of a stronger exercise partner encouraging the weaker exercise partner on exercise duration during an online exercise video game

TITLE

1a-i) Identify the mode of delivery in the title

yes. "online exercise video game"

1a-ii) Non-web-based components or important co-interventions in title

No. This was not really an intervention, but rather an efficacy study of the isolated effect of a superior virtual partner on effort during exercise. All non-web-based components were purely instructional (e.g., "please fill out this questionnaire", "your task today is to hold this exercise for as long as you can", etc.). It is our opinion that including this is the title would be unnecessary.

1a-iii) Primary condition or target group in the title

No. This was essentially a study of a basic psychological process (e.g., motivation gain), which is the key feature of the study. We felt it was desirable for us, then, to highlight this feature in the title, especially since it is not a RCT. We would be happy to revise, if needed, but for now we will stick with the current title and wait for further advisement.

ABSTRACT

1b-i) Key features/functionalities/components of the intervention and comparator in the METHODS section of the ABSTRACT

yes. "With the assistance of the experimenter, participants (n = 115) played an exercise video game, performing a series of five abdominal plank exercises where they were asked to hold the plank for as long as possible (Time 1). They were then randomized to a condition (Individual, Partner-without-encouragement, or Partner-with-encouragement,) where they performed the exercises again (Time 2)."

1b-ii) Level of human involvement in the METHODS section of the ABSTRACT

Yes. "With the assistance of the experimenter..."

1b-iii) Open vs. closed, web-based (self-assessment) vs. face-to-face assessments in the METHODS section of the ABSTRACT

yes. "Participants were recruited online and face-to-face from the campus of Michigan State University. With the assistance of the experimenter, participants (n = 115) played an exercise video game in a laboratory..."

1b-iv) RESULTS section in abstract must contain use data

Yes. "Those who exercised in online teams (n=80) exercised significantly longer (time = 78.8s, P < .001) than those who worked individually (n = 35). However, exercise duration was shorter when one's more capable partner gave verbal encouragement (n = 55) than when s/he did not (n = 25) (a mean difference of 31.14s)"

1b-v) CONCLUSIONS/DISCUSSION in abstract for negative trials

yes. "Exercising conjunctively with an internet partner can boost one's duration of exercise. However, encouragement from the stronger to the weaker member can mitigate these gains, especially if one perceives such comments being directed at someone other than themselves."

INTRODUCTION

2a-i) Problem and the type of system/solution

Yes. "Despite the links between regular physical activity and positive health benefits [1-7], less than 5% of US adults achieve recommended levels of intensity and duration [1-8]. Recent strategies to promote physical activity have harnessed the Internet, a medium through which the delivery of interventions can be highly cost-effective, with the potential to reach and impact a wide audience [10]. However, internet-delivered interventions typically produce only small effect-sizes [9] and, thus, may benefit from supplemental strategies to increase the intensity and duration of physical activity."

2a-ii) Scientific background, rationale: What is known about the (type of) system

Yes. "However, internet-delivered interventions typically produce only small effect-sizes [9] and, thus, may benefit from supplemental strategies to increase the intensity and duration of physical activity."

Justification: "Recent research has highlighted the influential role of social factors in physical activity behavior, including exercising with a partner [10-11]. When exercising under the right conditions, exercise partners have been shown to increase the intensity and duration of exercise by up to 208% [12-13]. Importantly, exercising with a partner affords the opportunity to encourage one another, which may further increase the duration of exercise. However, few, if any, internet-delivered interventions create opportunities for exercising in real-time with other exercise participants. "

METHODS

3a) CONSORT: Description of trial design (such as parallel, factorial) including allocation ratio

Objectives: "The purpose of the current study was to test the efficacy of an internet partner in increasing exercise duration. Specifically, we tested whether being the 'weak link' on a team with an internet partner could motivate one to exercise longer than when exercising alone. Further, we tested whether encouragement from the partner could motivate one to exercise longer, still. "

Hypotheses: "Although no previous studies on the Köhler effect have examined the influence of encouragement on the duration of exercise in the weak link, we reasonably hypothesized that encouragement would further boost exercise duration."

3b) CONSORT: Important changes to methods after trial commencement (such as eligibility criteria), with reasons

No methods were changed after commencement.

3b-i) Bug fixes, Downtimes, Content Changes

No. We don't believe this applies to our particular investigation, as the system was static throughout the entire study.

4a) CONSORT: Eligibility criteria for participants

Yes. "Eligible participants were students who had no physical injuries that would prevent or obstruct their performance during an isometric plank exercise. "

4a-i) Computer / Internet literacy

No. We feel that this was not relevant for our particular investigation. All computer related tasks were performed by the experimenter, except for filling out online questionnaires (which were completed in the presence/with the assistance of the experimenter in the laboratory). In either case, it was not addressed.

4a-ii) Open vs. closed, web-based vs. face-to-face assessments:

Yes. "Eligible participants were students who had no physical injuries that would prevent or obstruct their performance during an isometric plank exercise. "

4a-iii) Information giving during recruitment
Yes. "Students were recruited based on their interest in getting a good workout and were told that they would be playing an exercise video game and performing abdominal plank exercises for as long as they felt comfortable"
4b) CONSORT: Settings and locations where the data were collected
Yes. "The study was conducted in a laboratory on the campus of a large Midwestern university in the Departments of Kinesiology and Psychology."
4b-i) Report if outcomes were (self-)assessed through online questionnaires
This was not a 'web-based' trial, per se, but yes, online questionnaires were used, even though participants completed them in the laboratory.

"A baseline measure of self-efficacy was then taken using an online questionnaire completed in the laboratory"
4b-ii) Report how institutional affiliations are displayed
No. All participants were students enrolled at the university.
5) CONSORT: Describe the interventions for each group with sufficient details to allow replication, including how and when they were actually administered
5-i) Mention names, credential, affiliations of the developers, sponsors, and owners
All software was adapted from existing software for the purposes of this study. There were no conflicts of interest.

"Participants initially watched a brief instructional video from the PS2-Eye Toy Kinetic software in which a virtual trainer demonstrated the five exercises"
5-ii) Describe the history/development process
No. Again, we used existing software for a one-time laboratory trial, and adoption/use rates were not of interest.
5-iii) Revisions and updating
No. The intervention was static throughout the study.
5-iv) Quality assurance methods
Yes.

"Participants were asked throughout the session if they understood all instructions and, if not, the experimenter addressed his/her questions."

5-v) Ensure replicability by publishing the source code, and/or providing screenshots/screen-capture video, and/or providing flowcharts of the algorithms used
Yes. The relevant algorithm is the one used to calculate the virtual partner's exercise duration:

"That feedback score was manipulated to be 1.4 times the participant's own actual performance. "
5-vi) Digital preservation
No. There was no true online component. Only the perception that there was.
5-vii) Access
No. Again, there was no true online component/application (aside from online questionnaires, using Survey Monkey, which were not critical to the study).
5-viii) Mode of delivery, features/functionalities/components of the intervention and comparator, and the theoretical framework

Yes. This is all implicit in the exercise task and procedure sub-sections of the methods:

"Exercise Task
The task for this study was an abdominal plank exercise. These are body-weight resistance exercises where participants suspend their own body weight using their abdominal muscles. These exercises are also isometric in nature, require very little coordination, and are highly effort-based. Each exercise targeted the abdominal muscles, but there were slight differences between each (e.g., holding a push-up position on one's forearms vs. on each side; a detailed description of all exercises is provided elsewhere [12]).
The exercises were performed as part of an exercise video game designed for the Playstation 2 (PS2) gaming module as used in a previous study [12]. The software used was EyeToy: Kinetic, a game that offers a variety of fitness activities (e.g. yoga, strengthening exercises, combat exercises). This particular software operates in conjunction with an additional accessory called the Eye Toy, designed specifically for the PS2 system. The Eye Toy is a small camera that connects to the PS2 system via a USB cable and allows images of the user to be displayed on the TV monitor and interact with objects in a virtual environment supported by the software.
Procedure

A detailed description of experimental procedures is provided elsewhere [12]. In the current study, we simply describe the basic procedure and note how the encouragement manipulation was achieved.
After arriving at the laboratory, participants initially watched a brief instructional video from the PS2-Eye Toy Kinetic software in which a virtual trainer demonstrated the five exercises. A baseline measure of self-efficacy was then taken using an online questionnaire completed in the laboratory. Participants were asked throughout the session if they understood all instructions and, if not, the experimenter addressed his/her questions. All participants then performed the first block of exercises, holding each of the five exercises for as long as they could and with 30s rest periods between each exercise. Immediately after each exercise, the participant announced his/her perceived exertion. All participants were given veridical feedback on their performance (i.e., the average of the number of seconds they held each exercise).
The condition manipulation was introduced at this point. Participants in the individual control conditions simply rested for 10 min. Participants in the partner conditions were told that another participant was being run simultaneously at another lab on campus, and that the two participants would be able to see one another over an internet video connection during future trials. The participants then met briefly with that other, same-sex participant in a controlled Skype-like interaction (we will refer to that other participant hereafter as "the partner"). In reality, the partner was an experimental confederate whose side of the interaction was pre-recorded. After the interaction, participants were also given bogus feedback on how well the partner had done on the first trial. That feedback score was manipulated to be 1.4 times the participant's own actual performance. This discrepancy was chosen based on previous research that suggests that this moderate discrepancy leads to the greatest increases in exercise duration (i.e., the greatest Köhler effect) [18-19].
Participants were told that they and their partners would be a two-person exercise team. In the encouragement condition, both teammates were told that they would have the opportunity to communicate with each other during the exercises but that, due to technical problems, this capability would only be provided for the partner. Thus, ostensibly both could speak, but only the partner would be heard by the subject. No mention was made of any audio link between partners in the no-encouragement condition. For both partner conditions, it was further explained that they were working towards a team score, where the team score would be the persistence score of the first teammate to quit an exercise (i.e., as soon as either partner quit, the exercise was over). This task structure is more commonly known as a conjunctive task- when the group's performance is defined by the performance of the least-capable member (i.e., the 'weak link'). Following these instructions, all participants responded again to the self-efficacy measure.
Block 2 then commenced. In the individual control condition, the participants could only see him/herself on the screen, as during Block 1. In the partner conditions, the participant could see the partner's image (which was actually prerecorded) before and during the exercise; the participant believed that the partner could likewise see his/her (the participant's) image. The images available to the participant suggested that s/he was always the first to quit each exercise. The video link was allegedly frozen as soon as either teammate quit an exercise and until just before the start of the next exercise; hence, the participant only knew that his/her partner had been able to persist longer, but not just how much longer. In the encouragement condition, a pre-recorded series of phrases of encouragement was played through a set of computer speakers controlled by the experimenter. The phrases were audible approximately every 15s (+ 3s) and followed the following, fixed progression: "you can do it", "you got this", "keep it going", "you're doing good", "stay strong here", "give it your best". After Block 2 was over, the participant completed a series of questionnaires (self-efficacy, intention to exercise, enjoyment of physical activity, and manipulation checks). S/He was then debriefed, thanked, and excused."

5-ix) Describe use parameters

No. Participants were exposed to all stimulii at the same timing, frequency and intensity, as part of the experimental procedure (with differences between conditions, as described in the methods).

5-x) Clarify the level of human involvement

No. Human involvement is implicit, as the experimenter had to run the session.

5-xi) Report any prompts/reminders used

No. All participant activities were guided by the experimenter, who told them when to perform each activity.

5-xii) Describe any co-interventions (incl. training/support)

No. Not applicable.

6a) CONSORT: Completely defined pre-specified primary and secondary outcome measures, including how and when they were assessed

Yes.

"Duration of exercise. Duration of exercise was measured via the total number of seconds that the exercise was held. Block scores were calculated by taking the summed total of the five exercises within each trial.

Self-efficacy (SE). Task SE was measured with a scale developed specifically for this program of research. The measures contained five items, each corresponding to one of the five exercises within each trial. All items were preceded by the stem “What is the number of seconds that you are completely confident you can hold:” followed by “The first exercise”; “the second exercise” and so on for each of the five exercises. Respondents wrote in the number of seconds in a blank box following each item. The questionnaire was administered at three time points: once before Block 1 (after the participant had watched a brief instructional video demonstrating the exercises), a second time after Block 1 but before performing the five exercises at Block 2, and a third time after Block 2. A total SE score for each trial was calculated by taking the sum of the five items within each trial.

Ratings of perceived exertion (RPE). RPE was measured using the 6-20 version of the Borg RPE scale [20]. The scale ranges from 6-20 where 6 means “no exertion at all” and 20 means “maximal exertion.” Participants were asked to rate their exertion at the end of each exercise, with particular reference to their perceived exertion at the moment right before the end of the exercise.

Task Enjoyment. Task enjoyment was measured using a short 8-item version of the Physical Activity Enjoyment Scale (PAES) [21]. Each item was rated on a 7-point bipolar scale beginning with the stem “Please rate how you feel at the moment about the physical activity you have been doing according to the following scales” (e.g., 1= “I loved it”; 7 = “I hated it”). Previous studies have shown high correlations with the longer, 18-item scale (r = .94) [22] and strong reliability (alpha = .91) [23].

Intention to exercise. Adapted from another measure [24], Intention was assessed with a single item, “I intend to exercise tomorrow for at least 30 minutes” on a scale of -3 (“Not at all true for me”) to +3 (“Completely true for me”).

Post-experimental questionnaire. Besides some questions checking participants’ understanding of the instructions and procedures, there were questions probing their perceived task ability, a rating of task difficulty, and a rating of effort expended on the task, each made on 8-point scales. Participants in the partner conditions were also asked to rate their partner’s relative ability on a 9-point scale (where 1=I am much more capable and 9=my partner is much more capable). "

Timing of each measure is detailed throughout the procedure section.

6a-i) Online questionnaires: describe if they were validated for online use and apply CHERRIES items to describe how the questionnaires were designed/deployed

No. The fact that we used online questionnaires was not critical to this study. Participants completed online questionnaires in the laboratory. We used them simply for the sake of convenience. Therefore, we do not report on this item.

6a-ii) Describe whether and how “use” (including intensity of use/dosage) was defined/measured/monitored

Not applicable.

6a-iii) Describe whether, how, and when qualitative feedback from participants was obtained

Not obtained/applicable.

6b) CONSORT: Any changes to trial outcomes after the trial commenced, with reasons

No. No changes.

7a) CONSORT: How sample size was determined

7a-i) Describe whether and how expected attrition was taken into account when calculating the sample size

No. We did not take into account attrition simply because, given the nature of the research question, we recruited and tested until we met sample size requirements for observing the effect we hoped to achieve.

7b) CONSORT: When applicable, explanation of any interim analyses and stopping guidelines

No. Not applicable.

8a) CONSORT: Method used to generate the random allocation sequence

yes.

"participants were randomly assigned to conditions within each wave using a randomization function on Microsoft Excel"

8b) CONSORT: Type of randomisation; details of any restriction (such as blocking and block size)

Yes.
"...participants were randomly assigned to conditions..."

9) CONSORT: Mechanism used to implement the random allocation sequence (such as sequentially numbered containers), describing any steps taken to conceal the sequence until interventions were assigned

No. not applicable.

10) CONSORT: Who generated the random allocation sequence, who enrolled participants, and who assigned participants to interventions

Yes.

"participants were randomly assigned to conditions within each wave using a randomization function on Microsoft Excel generated by one of the primary investigators"

11a) CONSORT: Blinding - If done, who was blinded after assignment to interventions (for example, participants, care providers, those assessing outcomes) and how

11a-i) Specify who was blinded, and who wasn’t

Yes.
"participants were randomly assigned to conditions (single blind) within each wave using a randomization function on Microsoft Excel generated by one of the primary investigators"

11a-ii) Discuss e.g., whether participants knew which intervention was the “intervention of interest” and which one was the “comparator”

Yes.

"participants were randomly assigned to conditions (single blind) within each wave using a randomization function on Microsoft Excel generated by one of the primary investigators"

11b) CONSORT: If relevant, description of the similarity of interventions

not relevant.

12a) CONSORT: Statistical methods used to compare groups for primary and secondary outcomes

Yes.

"Following the methods of analyses done in earlier studies, exercise duration was taken as the sum of the time each participant held the five exercises within each block, producing a Block 1 and Block 2 score. There are, of course, individual differences in fitness, intrinsic task interest, and strength that we wished to control for. This can be done in different ways. In many prior studies [14, 25], participants' Block 1 performance has been used as a baseline and difference scores (i.e., Block 2 – Block 1) were the primary dependent variable, one that expressed each participant's Block 2 score relative to his/her Block 1 score (the latter baseline score reflected individual differences in fitness and strength). An alternative, less vulnerable to certain problems that can arise from the use of difference scores [e.g., 26], is to use Block 1 scores as a covariate in the analysis of Block 2 scores. Here, we present the results using the former, difference-score method because the mean values presented for such an analysis are more directly understood and interpreted than means adjusted for a covariate. But the reader should note that an identical pattern of results is obtained with either method.
The analyses of the exercise duration data was to proceed in two stages. The first was to check whether there were any history or cohort effects attributable to the interval between the two data collection waves. It employed a 2 (Condition: Individual vs. partner-without-encouragement) x 2 (Sex) x 2 (Data collection wave: Early vs. Late) ANOVA on exercise duration difference scores (i.e., Trial 2 duration – Trial 1). Although overall sex differences in the magnitude of the Köhler effect are rare, some interesting sex effects have been reported [e.g., 25]; hence participant sex was included as an experimental factor. This stage 1 analysis would permit both confirming with the larger data set the Köhler effect originally reported in Kerr et al. [17], and checking whether its magnitude differed between the participants drawn from Kerr et al. [17] and those newly recruited in the same experimental conditions for the present study. The second stage was designed to examine the primary question addressed by this paper—would verbal encouragement to the weaker partner alter the Köhler effect. If there were no data-wave moderation effects, as anticipated, we planned to drop the data-wave factor and add the no-encouragement condition in a 3 (Condition: Individuals, Partner-without-encouragement, Partner-with-encouragement) x 2 (Sex) ANOVA on exercise duration difference scores.
Although our primary focus was on exercise duration, we also examined several other variables, primarily to determine if encouragement altered any of the effects previously observed [12,17] when no encouragement was provided. For those variables collected at the end of the study (viz., task effort, intent to exercise, own task ability), the analyses would employ 3 (Condition) x 2 (Sex) analyses of variance; since the individual participants had no partner, this became a 2 (Condition: Encouragement vs No-encouragement) x 2 (Sex) ANOVA for ratings of own ability relative to one's partner. For those variables (viz., perceived effort, self efficacy) collected during the exercise trials, a within-Ss Block factor was added to the ANOVA. Finally, a covariate (viz., the pre-exercise estimate of task self-efficacy) was included in the analysis of self efficacy.
"

12a-i) Imputation techniques to deal with attrition / missing values

There was no attrition, so this is not reported.

There were no missing data on the primary outcome, so this is not reported.

12b) CONSORT: Methods for additional analyses, such as subgroup analyses and adjusted analyses

none.

RESULTS

13a) CONSORT: For each group, the numbers of participants who were randomly assigned, received intended treatment, and were analysed for the primary outcome

yes. it is included in the participant flow chart.

13b) CONSORT: For each group, losses and exclusions after randomisation, together with reasons

yes. in flow diagram.

13b-i) Attrition diagram

yes. in flow diagram

14a) CONSORT: Dates defining the periods of recruitment and follow-up

yes. in flow diagram

14a-i) Indicate if critical "secular events" fell into the study period

no/not applicable (study was a series of one-time, hour long sessions where the outcomes should have very little correlation with secular events.

14b) CONSORT: Why the trial ended or was stopped (early)

not applicable. did not stop early

15) CONSORT: A table showing baseline demographic and clinical characteristics for each group

no. included in the text as to not take up unnecessary space.

"Overall, the average participant was a sophomore/junior (mean of 2.45 where 1=1st year, 2=2nd year, etc.) aged 20.3 years (SD=3.3)."

15-i) Report demographics associated with digital divide issues

not aside from age. digital divide would likely have very little influence on the primary outcome, as computer literacy would have little to do with how the respond to a virtually present person (virtually present people are ubiquitous- e.g., via telephones, televisions, email, etc.). In any case, we did not directly assess this.

""Overall, the average participant was a sophomore/junior (mean of 2.45 where 1=1st year, 2=2nd year, etc.) aged 20.3 years (SD=3.3).""

16a) CONSORT: For each group, number of participants (denominator) included in each analysis and whether the analysis was by original assigned groups

16-i) Report multiple "denominators" and provide definitions

Yes. Indicated in flow chart/degrees of freedom.

16-ii) Primary analysis should be intent-to-treat

Not applicable. All participants were 'treated'.

17a) CONSORT: For each primary and secondary outcome, results for each group, and the estimated effect size and its precision (such as 95% confidence interval)

I'm not sure that this (or many of the items in this checklist) is relevant for our study. Please advise.

17a-i) Presentation of process outcomes such as metrics of use and intensity of use

no. not relevant.

17b) CONSORT: For binary outcomes, presentation of both absolute and relative effect sizes is recommended

not relevant.

18) CONSORT: Results of any other analyses performed, including subgroup analyses and adjusted analyses, distinguishing pre-specified from exploratory

not applicable. we did not conduct any other analyses.

18-i) Subgroup analysis of comparing only users

not applicable. we did not conduct subgroup analyses.

19) CONSORT: All important harms or unintended effects in each group

not applicable. none were harmed and there were no unintended effects.

19-i) Include privacy breaches, technical problems

not applicable. no privacy breaches or technical problems.

19-ii) Include qualitative feedback from participants or observations from staff/researchers

No. Although we did collect such data (open ended questions in post-experiment survey as part of a manipulation check), we have chosen not to report it only because we did not find anything out of the ordinary. Had we found that participants were reporting on anything suggesting that the manipulation was not successful, we would report it.

DISCUSSION

20) CONSORT: Trial limitations, addressing sources of potential bias, imprecision, multiplicity of analyses

20-i) Typical limitations in ehealth trials

"Of course, this study has its limitations. The study was conducted in a highly controlled laboratory setting, and it may be premature to suggest that these findings generalize to free-living conditions and other populations who are more physically inactive. Second, although we found that being the weak link can motivate participants to exercise longer, it is not clear if the same strategy could have the same positive impact on other dimensions of physical activity behavior (e.g., frequency and intensity of exercise). Lastly, we tested participants in only one session of exercise, and repeatedly being the 'weak link' over several sessions may actually be de-motivating [34]. We should note, however, that some of the potential limitations have been addressed in other studies. For instance, researchers have recently observed the effort-boosting effect of a superior partner in other physical activity tasks and conditions, such as duration of aerobic exercise [13] and performance in swimming relay competitions, respectively [35]. "

21) CONSORT: Generalisability (external validity, applicability) of the trial findings

21-i) Generalizability to other populations

"The study was conducted in a highly controlled laboratory setting, and it may be premature to suggest that these findings generalize to free-living conditions and other populations who are more physically inactive"

21-ii) Discuss if there were elements in the RCT that would be different in a routine application setting

Not applicable. It was not an RCT.

22) CONSORT: Interpretation consistent with results, balancing benefits and harms, and considering other relevant evidence

22-i) Restate study questions and summarize the answers suggested by the data, starting with primary outcomes and process outcomes (use)

The purpose of this study was to test the efficacy of a superior internet partner (with and without encouragement) as a strategy for increasing the duration of exercise. Consistent with previous studies [12,13], after controlling for individual differences in strength, participants who simply exercised with a partner as the weak link persisted longer (on average, 78.8s) than those who exercised by themselves, a gain of 33.6%.

22-ii) Highlight unanswered new questions, suggest future research

yes.

"The study was conducted in a highly controlled laboratory setting, and it may be premature to suggest that these findings generalize to free-living conditions and other populations who are more physically inactive. Second, although we found that being the weak link can motivate participants to exercise longer, it is not clear if the same strategy could have the same positive impact on other dimensions of physical activity behavior (e.g., frequency and intensity of exercise). Lastly, we tested participants in only one session of exercise, and repeatedly being the 'weak link' over several sessions may actually be de-motivating [34]."

"Future research should examine the effects of a wider range of messages, exercise tasks, and conditions to help inform the design of group-based, electronically-mediated physical activity interventions. "

Other information

23) CONSORT: Registration number and name of trial registry

No. It's not registered.

24) CONSORT: Where the full trial protocol can be accessed, if available

yes. The protocol is described in detail in the methods section, which I will not copy and paste here for the sake of the reader's time.

25) CONSORT: Sources of funding and other support (such as supply of drugs), role of funders

"Support for this research was provided by the Robert Wood Johnson Foundation's Pioneer Portfolio through a grant from its national program, "Health Games Research: Advancing Effectiveness of Interactive Games for Health.""

X26-i) Comment on ethics committee approval

"The study was conducted in a laboratory on the campus of Michigan State University in the Departments of Kinesiology and Psychology and was approved by the IRB"

x26-ii) Outline informed consent procedures

Yes.

"After arriving at the laboratory and obtaining participant consent, participants initially watched a brief instructional video ...."

X26-iii) Safety and security procedures

"After arriving at the laboratory, participant consent was obtained and all were ensured of the confidentiality and voluntary nature of their participation"

X27-i) State the relation of the study team towards the system being evaluated

"The authors have no conflicts of interest to report."
